# Analyzing the risk factors of unilateral trigeminal neuralgia under neurovascular compression

**DOI:** 10.3389/fnhum.2024.1349186

**Published:** 2024-04-17

**Authors:** Juncheng Yan, Luoyu Wang, Lei Pan, Haiqi Ye, Xiaofen Zhu, Qi Feng, Zhongxiang Ding, Xiuhong Ge, Lei Shi

**Affiliations:** ^1^Department of Rehabilitation, Hangzhou First People's Hospital, Hangzhou, China; ^2^Department of Radiology, Hangzhou First People's Hospital, Hangzhou, China; ^3^The Cancer Hospital of the University of Chinese Academy of Sciences (Zhejiang Cancer Hospital), Institute of Basic Medicine and Cancer (IBMC), Hangzhou, China

**Keywords:** trigeminal neuralgia, risk factors, machine learning, neurovascular compression, magnetic resonance imaging

## Abstract

**Background:**

This study aimed to explore the risk factors and potential causes of unilateral classical or idiopathic trigeminal neuralgia (C-ITN) by comparing patients and healthy controls (HCs) with neurovascular compression (NVC) using machine learning (ML).

**Methods:**

A total of 84 C-ITN patients and 78 age- and sex-matched HCs were enrolled. We assessed the trigeminal pons angle and identified the compressing vessels and their location and severity. Machine learning was employed to analyze the cisternal segment of the trigeminal nerve (CN V).

**Results:**

Among the C-ITN patients, 53 had NVC on the unaffected side, while 25 HCs exhibited bilateral NVC, and 24 HCs showed unilateral NVC. By comparing the cisternal segment of CN V between C-ITN patients on the affected side and HCs with NVC, we identified the side of NVC, the compressing vessel, and certain texture features as risk factors for C-ITN. Additionally, four texture features differed in the structure of the cisternal segment of CN V between C-ITN patients on the unaffected side and HCs with NVC.

**Conclusion:**

Our findings suggest that the side of NVC, the compressing vessel, and the microstructure of the cisternal segment of CN V are associated with the risk of C-ITN. Furthermore, microstructural changes observed in the cisternal segment of CN V on the unaffected side of C-ITN patients with NVC indicate possible indirect effects on the CN V to some extent.

## Introduction

Trigeminal neuralgia (TN) is a chronic condition characterized by sudden, brief electric shock-like pain. These pain episodes typically last from seconds to a couple of minutes, with some enduring slightly longer durations (Leal et al., 2019; Cruccu et al., 2020; Danyluk et al., 2021). TN often affects one or more branches of the trigeminal nerve (CN V), particularly the maxillary (V2) and mandibular (V3) branches (Tohyama et al., 2020; Zhang et al., 2020). Pain episodes are frequently triggered by innocuous daily activities such as talking, chewing, or tooth brushing. While TN predominantly manifests as a unilateral condition, bilateral TN cases are occasionally reported (Cruccu et al., 2020). TN has an annual incidence of ~4–13 per 100,000 individuals, with a higher prevalence among elderly women (Zeng et al., 2021). The condition can result in significant psychological distress, including anxiety, depression, and, in severe cases, suicidal thoughts (Cruccu et al., 2020; Tohyama et al., 2020; Zhang et al., 2020).

TN is categorized into classical TN (CTN), secondary TN (STN), and idiopathic TN (ITN), with CTN being the most prevalent, accounting for ~75% of all cases (Cruccu et al., 2020). Neurovascular compression (NVC) is widely recognized as the primary cause of CTN (Cruccu et al., 2020). However, studies have indicated that NVC can also be present in asymptomatic people and in the unaffected side of CTN patients (Adamczyk et al., 2007; Hardaway et al., 2019; Araya et al., 2020; Arda et al., 2021; Zhao et al., 2022).

According to the International Classification of Headache Disorders, 3rd edition (ICHD-3; Headache Classification Committee of the International Headache Society, 2018), patients with NVC but with no morphological changes in the nerve root are classified as having ITN. We hypothesize that NVC may underlie the pathogenesis of these cases of ITN, which we refer to in this study as ITN with NVC. NVC can result in macrostructural alterations such as distortion, displacement, and thinning of the trigeminal nerve at the compression site (Hao et al., 2020; Inoue et al., 2021), as well as microstructural changes including local degeneration and demyelination (Moon et al., 2018; Wang et al., 2019; Bendtsen et al., 2020; Cruccu et al., 2020; Tohyama et al., 2020; Zhang et al., 2020). In our previous research, we had observed microstructural changes in the affected side of the CN V (Ge et al., 2022), but we did not compare these changes with those observed in healthy controls (HCs) with NVC.

In this study, we utilize machine learning (ML) to compare the CN V of individuals with unilateral CTN or ITN and NVC (C-ITN) with that of HCs and explore the microstructural differences between the affected or unaffected side of the C-ITN and HCs, both with NVC. We hypothesize that the microstructure may be the risk factor for NVC in C-ITN patients and could help clarify the pathogenesis of C-ITN.

## Methods and materials

### Study design and subjects

Subjects were recruited from Hangzhou First People's Hospital between July 2021 and August 2022, provided they met the inclusion and exclusion criteria. This study received approval from the ethics committee of Hangzhou First People's Hospital (IRB# No. 202107002). All subjects provided informed consent.

The inclusion criteria for patients were as follows: (1) diagnosis of CTN or ITN with NVC according to ICHD-III criteria (Headache Classification Committee of the International Headache Society, 2018; Ge et al., 2022); (2) patients with unilateral CTN in the distribution of one or more branches of the trigeminal nerve; (3) conventional magnetic resonance imaging (MRI) T1WI and T2WI sequence examinations revealing no evident abnormal brain signals; (4) patients with good physical condition and willingness to cooperate with the MRI; (5) patients aged 20–75 years; (6) patients who are right-handed. The exclusion criteria were as follows: (1) patients with C-ITN who underwent surgical treatment; (2) patients with STN or ITN without NVC; (3) contraindications for MRI; (4) poor image quality affecting the analysis; (5) severe intracranial organic disease; (6) those who were left-handed.

A total of 78 age- and sex-matched HCs were recruited. The inclusion criteria were as follows: (1) patients with good basic physical condition and ability to cooperate with MRI and (2) those aged 20–75 years. The exclusion criteria were as follows: (1) TN; (2) contraindications for MRI; (3) poor image quality affecting the analysis; and (4) severe intracranial organic disease.

### Image acquisition

All patients and HCs underwent MRI using a 3.0T MRI scanner (Siemens, MAGNETOM Verio, Germany) and an eight-channel phased-array head coil. Patients completed an MRI scan sequence comprising T1-weighted imaging (T1WI), T2-weighted imaging (T2WI), 3D volume interpolation body part examination (3D-VIBE), and 3D short-time inversion recovery (3D-STIR) data acquisition, as detailed in our previous study (Ge et al., 2022). T1WI parameters were as follows: TR = 1,500 ms; TE = 8.5 ms; flip angle = 150°; FOV = 230 × 230 mm^2^; voxel size = 1.5 × 0.9 × 5.0 mm^3^; slice thickness = 5 mm; 20 slices. T2WI parameters were as follows: TR = 3,700 ms; TE = 96 ms; flip angle = 150°; FOV = 230 × 230 mm^2^; voxel size = 0.7 × 0.7 × 5.0 mm^3^; slice thickness = 5 mm; 20 slices. 3D-VIBE parameters were as follows: TR = 10 ms; TE = 3.69 ms; flip angle = 12°; FOV = 220 × 220 mm^2^; voxel size = 0.8 × 0.8 × 0.8 mm^3^; slice thickness = 0.8 mm; 60 slices. 3D-STIR parameters were as follows: SPC sequence; TR = 3,800 ms; TE = 194 ms; FOV = 230 × 230 mm^2^; voxel size = 0.9 × 0.9 × 0.9 mm^3^; slice thickness = 0.9 mm; 64 slices.

### Trigeminal never structure

**Figure 1:**
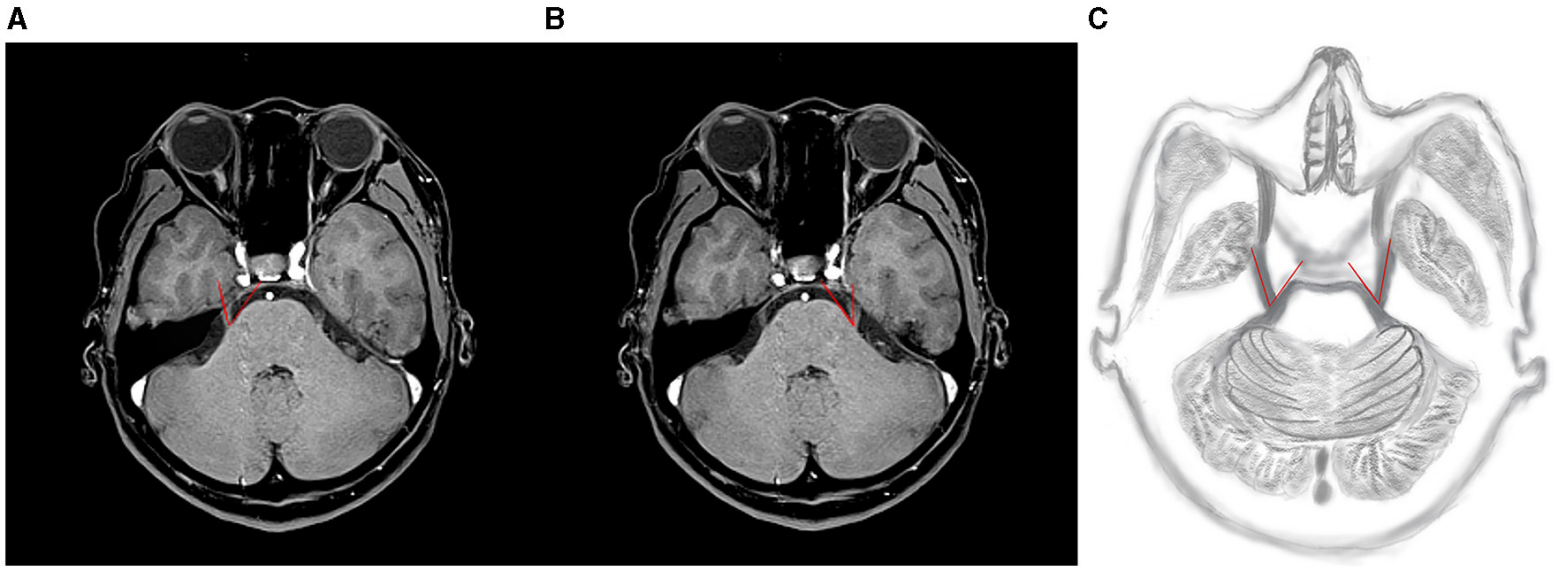
Example of TPA measurement: along the main axis of the trigeminal nerve, the point extending from the brainstem through the trigeminal nerve was tangential to the pons, forming an angle composed of two lines. **(A, B)** Illustrate TPA measurements on the right and left sides of C-ITN, respectively. **(C)** Shows a hand-drawn representation. TPA refers to the trigeminal pons angle, and C-ITN indicates classical or idiopathic trigeminal neuralgia with neurovascular compression.

Original images of 3D-VIBE and 3D-STIR sequences were sent to a Siemens postprocessing workstation for analysis. They were separately analyzed by a junior physician with 5 years of experience and a senior physician with 10 years of experience, both experienced in analyzing the trigeminal nerve structure. Trigeminal nerve structure measurements were conducted as described in our previous study (Ge et al., 2022). The trigeminal pons angle (TPA) was defined as the angle along the main axis of the CN V, with one point extending from the brainstem through CN V tangential to the pons, composed of two lines (Barzaghi et al., 2020; Ge et al., 2022) (Figure 1). The offending vessel, the position of offending vessel compression (POfvC), and the degree of offending vessel compression (DOfvC) were assessed. “Posterior” POfvC means that the offending vessel was located in the region along the cisternal segment of CN V near one-third of the length of the brainstem. “Anterior” POfvC means that the offending vessel is located in the region along the cisternal segment of CN V distal two-thirds of the length of the brainstem. DOfvC was categorized into four classes: class 0, no discernible relationship between the nerve and vessel was found or the relationship was unclear and difficult to assess; class 1, the vessel crossed or touched the nerve without a visible cerebrospinal fluid layer or root deformity; class 2, significant root indentation due to vessel compression was observed; and class 3, nerve distortion and/or displacement occurred (Hao et al., 2020; Ge et al., 2022).

### Radiomics analysis

**Figure 2:**
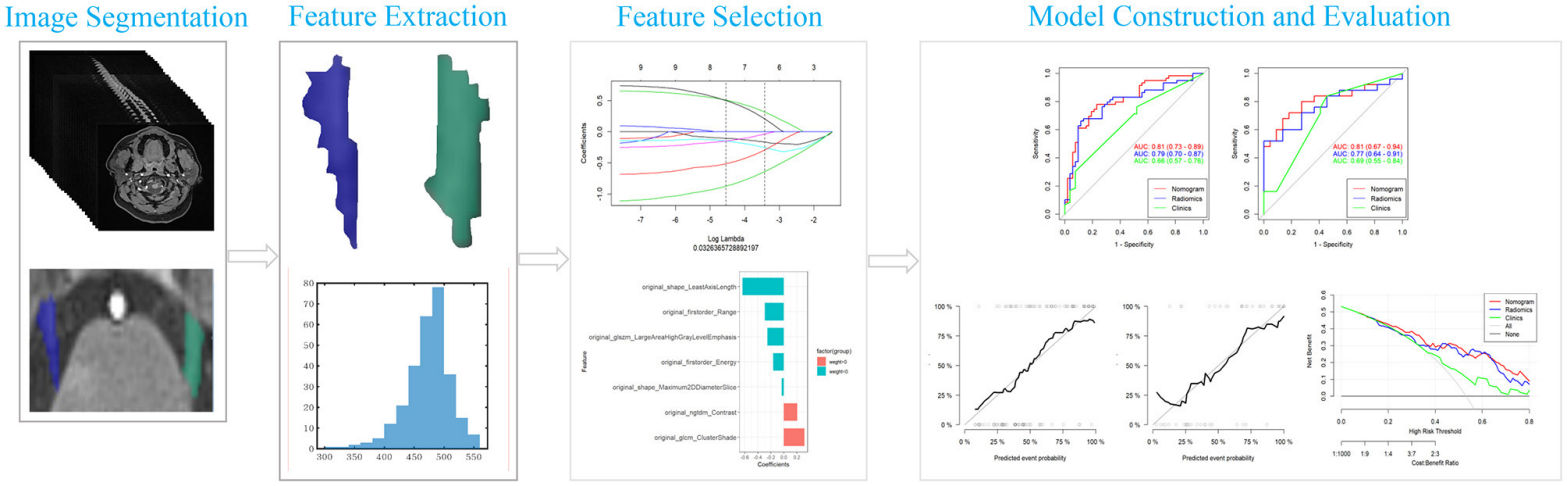
Flowchart of radiomics analysis. LASSO, least absolute shrinkage and selection operator; ROC, receiver operating characteristic curve.

The 3D-VIBE images and clinical parameters were uploaded to the uAI Research Portal (United Imaging Intelligence, China) and integrated into the widely used PyRadiomics package (https://pyradiomics.readthedocs.io/en/latest/index.html). To mitigate sample bias in grouping, a 10-fold cross-validation method was employed for the study population (Figure 2).

#### Delineation of the region of interest

The region of interest (ROI) was delineated along the edge of the trigeminal nerve, encompassing the cisternal segment of CN V (from the location where the nerve exits the pons to a predetermined boundary at the entrance of the Meckel's cave), and manually delineated slice by slice on 3D-VIBE images, excluding the offending vessel.

#### Data grouping

The data were randomly split into a training cohort (70%) and a validation cohort (30%).

#### Feature extraction

Features were extracted from the original ROI, including first-order statistics, shape and size, gray-level co-occurrence matrix, gray-level run-length matrix, gray-level size-zone matrix, gray-level dependence matrix, neighboring gray-tone difference matrix, and imaging filters.

#### Feature selection

Two feature selection methods, minimum redundancy maximum relevance (*mRMR*), and least absolute shrinkage and selection operator regression (LASSO) were used. Initially, *mRMR* was employed to eliminate redundant and irrelevant features, resulting in 10 retained features. LASSO was then applied to select the regular parameter λ, determining the number of features. Once the number of features was established, the most predictive subset was chosen, and corresponding coefficients were evaluated.

#### Model construction

Rad_score was computed as the sum of selected features, weighted by their coefficients. Rad_scores of the training and validation groups were compared. Subsequently, the radiomics score was combined with independent clinical–radiological predictors to construct a comprehensive nomogram using logistic regression.

#### Model evaluation

Model discrimination was quantified using the area under the curve (AUC) of the receiver operating characteristic (ROC). Calibration curves were used to assess the coincidence between the prediction model and actual outcomes. Decision curves were employed to visualize the clinical net benefit of prediction models.

### Statistical analysis

All statistical analyses were conducted using SPSS 26.0 and R software. Clinical and MRI morphological features were evaluated using the chi-squared test for nominal variables and the Wilcoxon test for continuous variables. Characteristics with a significance level of P of < 0.1 were further analyzed using univariate logistic regression to identify risk factors. Additionally, independent risk factors and the optimal rad_score were analyzed using multifactorial logistic regression to construct the prediction nomogram. Collinearity was assessed using the variance inflation factor (VIF), and features with VIF > 10 were removed. Delineation consistency between the junior and senior physicians was assessed using the intraclass correlation coefficient (ICC).

## Results

### Clinical features of C-ITN and HCs

**Figure 3:**
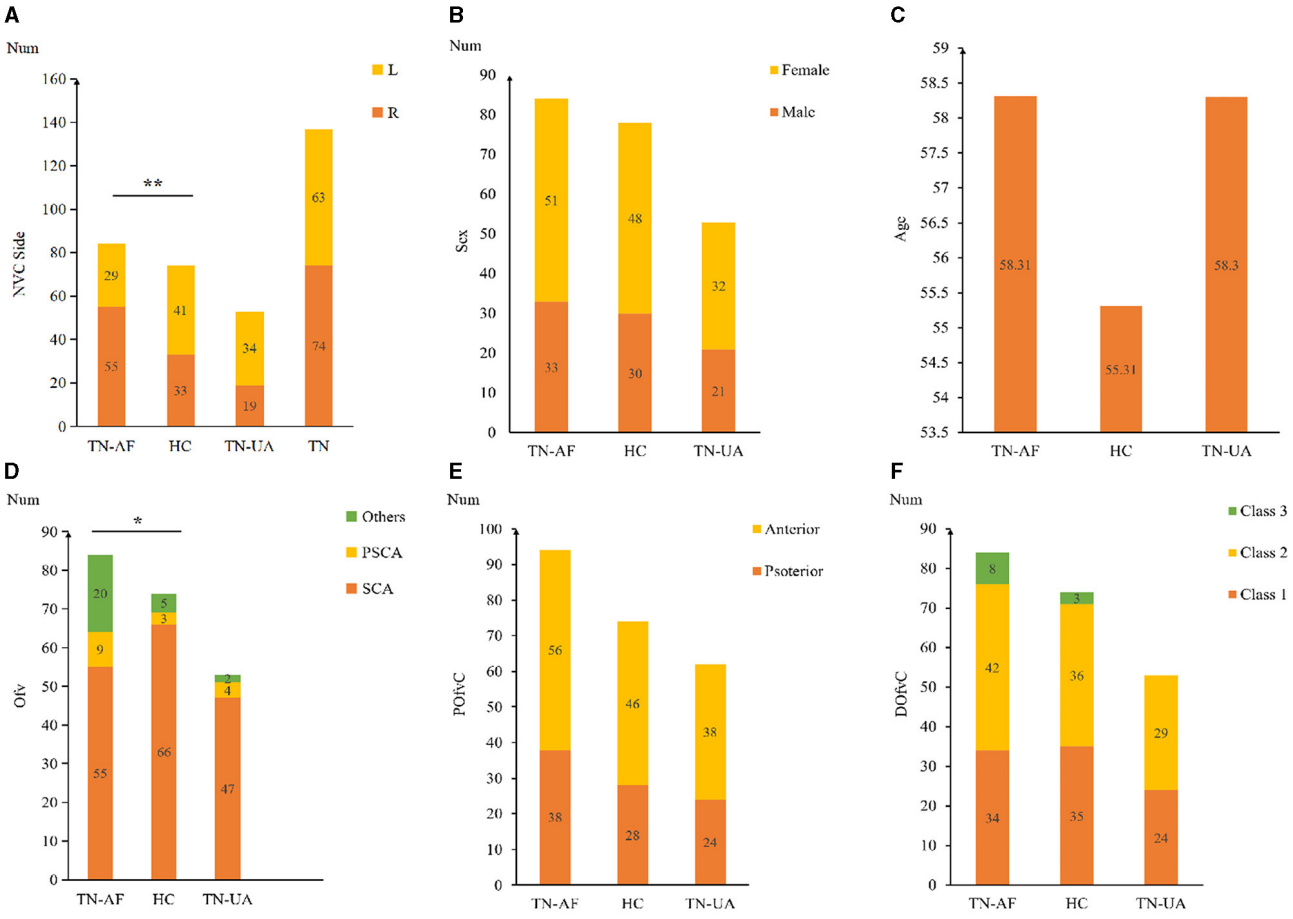
General information of 84 C-ITN patients and 78 HCs with NVC: **(A)** Shows the NVC side and numbers of TN-AF, HC, TN-UA, and TN. **(B)** Displays the gender distribution and numbers of TN-AF, HC, and TN-UA with NVC. **(C)** Presents the mean age of TN-AF, HC, and TN-UA with NVC. **(D)** Illustrates the Ofv and numbers of TN-AF, HC, and TN-UA with NVC. **(E)** Depicts the position of the POfvC and numbers of TN-AF, HC, and TN-UA. **(F)** Shows the DOfvC and numbers of TN-AF, CON, and TN-UA. TN-AF refers to the affected side of C-ITN, HCs denote healthy controls with NVC, TN-UA indicates the unaffected side of C-ITN with NVC, TN represents C-ITN patients with NVC, and NVC stands for neurovascular compression. Additionally, Ofv signifies the offending vessel, with PSCA referring to the posterior superior cerebellar artery and SCA indicating the superior cerebellar artery. DOfvC represents the degree of offending vessel compression, and POfvC denotes the position of offending vessel compression. * indicates *p* < 0.05, and ** indicates *p* < 0.01.

The general characteristics of 84 C-ITN patients and 78 HCs are presented in Figure 3. Among the C-ITN group patients, the left-to-right ratio was 29:545, with 53 patients exhibiting NVC on the unaffected side. In the HC group, 25 individuals had bilateral NVC, 24 had unilateral NVC, and 29 had no NVC. The main offending vessels in C-ITN on the affected side were the superior cerebellar artery (SCA) and posterior superior cerebellar artery (PSCA). Other offending vessels on the affected side included the following: posterior inferior cerebellar artery (PICA; two cases), vertebral artery (VA; two cases), basilar artery (BA; one case), SCA and PSCA (four cases), SCA and PICA (three cases), SCA and tiny vessels that are difficult to identify (TVHI; one case), SCA and VA (one case), and TVHI (six cases). Other offending vessels in HCs with NVC included the following: PICA (one case), VA (two cases), and TVHI (two cases). Other offending vessels in C-ITN with NVC on the unaffected side included the following: SCA and PSCA (two cases). Statistically significant differences were observed in the NVC side (*P* < 0.01) and offending vessel (*P* < 0.05) between the affected side of C-ITN and HCs with NVC.

### Intraclass correlation coefficient

According to ICC analysis, there was good agreement (ICC ≥ 0.75) for image features, TPA, offending vessel, POfvC, and DOfvC. The results delineated by the senior physician were selected for further analysis.

### The affected side of C-ITN patients and HCs with NVC

#### Feature extraction

**Figure 4:**
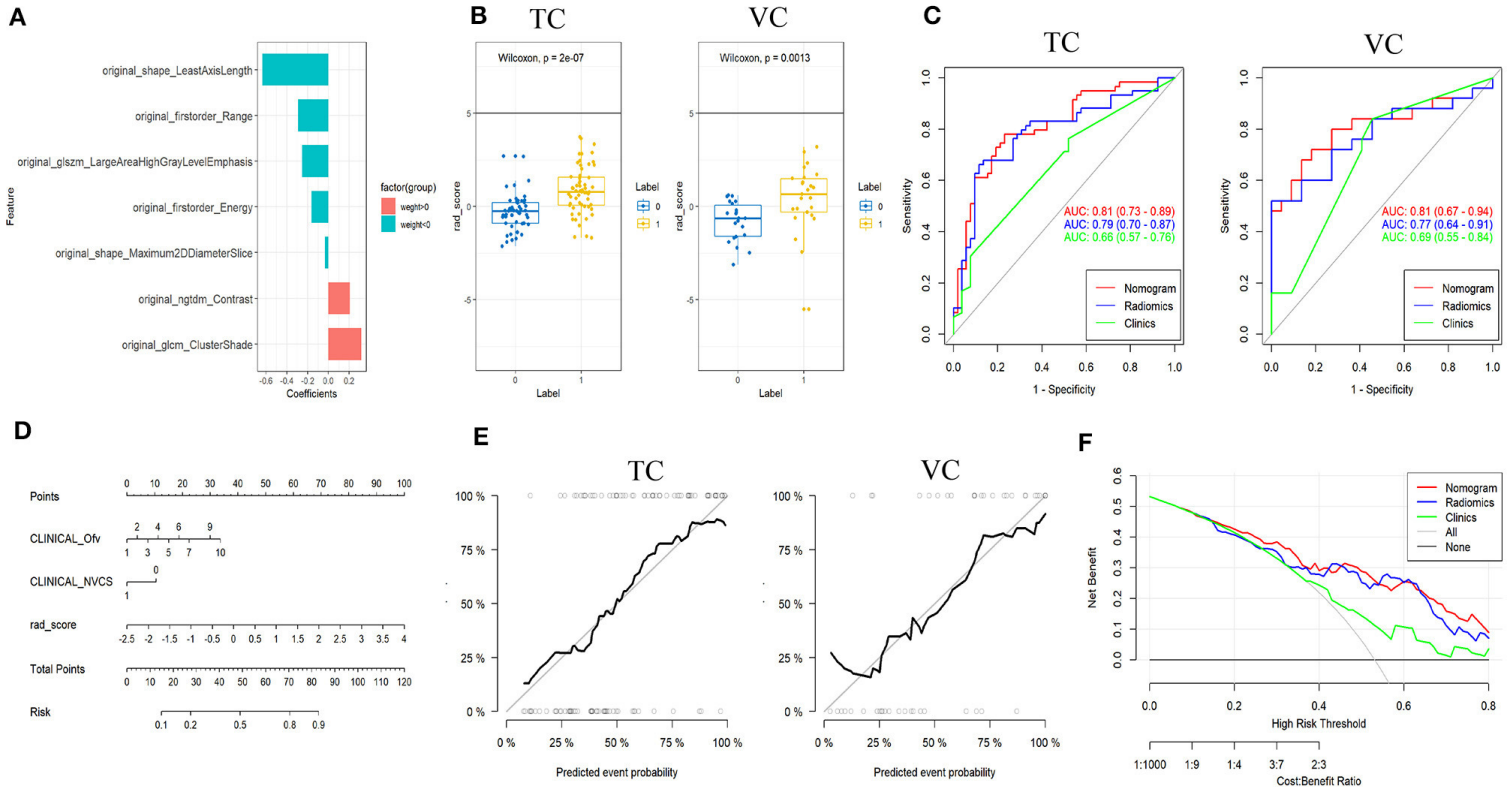
Results of patients with C-ITN on the affected side and HCs with 74 sides with NVC analyzed by machine learning. **(A)** Selected features and their importance ratio. **(B)** The Wilcoxon test of rad_score in the TC and VC. **(C)** ROC curves of the three models for the TC and VC. **(D)** Nomogram. **(E)** Calibration curves for the TC and VC. **(F)** Decision curve. C-ITN, classical trigeminal neuralgia or idiopathic trigeminal neuralgia with neurovascular compression; NVcs, cisternal segment of CN V; TC, training cohort; VC, validation cohort; ROC, receiver operating characteristic curve. 1, 2, 3, 4, 5, 6, 7, and 10 of the INCLINICAL_Ofv represent the superior cerebellar artery, anterior superior cerebellar artery, anterior inferior cerebellar artery, vertebral artery, basilar artery, superior cerebellar artery and anterior superior cerebellar artery, superior cerebellar artery and anterior inferior cerebellar artery, tiny vessels that are difficult to identify, respectively; 0, 1 of the INCLINICAL_NVC represent without and with NVC; Ofv, Offending Vessel; NVC, Neurovascular Compression.

A total of 117 features were selected for each ROI of C-ITN patients and HCs. After dimensionality reduction, seven textural features with greater weight were chosen, namely, least axis length (LAL), cluster shade (CS), range, large area high gray-level emphasis (LAHGLE), contrast, energy, and maximum 2D diameter slice (Figure 4).

#### Model construction and evaluation of the rad_score

In both the training and validation cohorts, the Wilcoxon test for rad_score showed a statistically significant difference (*P* < 0.05). Both cohorts exhibited consistent AUC values, indicating a good model fit (Figure 4).

#### Model construction of nomogram and model evaluation

The nomogram model was built using logistic regression for the rad_score combined with clinical characteristics. ROC curves displayed differences between various nomograms and clinical models, indicating superior performance of the nomogram model compared to the simple rad_score model and the clinical characteristics model (Figure 4 and Table 1). A simplified visual representation of the complex regression equation was created after constructing the nomogram. Calibration curves in both the training and validation cohorts demonstrated high diagnostic accuracy of the model (*P* = 0.10 vs. *P* = 0.92). Additionally, a decision curve was employed to assess the clinical utility of the model.

**Table 1:** The parameters of the three models.

**Model**		**AUC**	**Accuracy (%)**	**Sensitivity (%)**	**Specificity (%)**	**Pos. pred. value (%)**	**Neg. pred. value (%)**
Radiomics	TC	0.79	76.58%	66.10%	88.46%	86.67%	69.70%
	VC	0.77	74.47%	52.00%	100.00%	100.00%	64.71%
Clinics	TC	0.66	63.06%	76.27%	48.08%	62.50%	64.10%
	VC	0.69	70.21%	84.00%	54.55%	67.74%	75.00%
Nomogram	TC	0.81	77.48%	77.97%	76.92%	79.31%	75.47%
	VC	0.81	76.60%	85.00%	70.37%	68.00%	86.36%

TC, training cohort; VC, validation cohort; AUC, area under the curve.

### The unaffected side of C-ITN and HCs, both with NVC

#### Feature extraction

**Figure 5:**
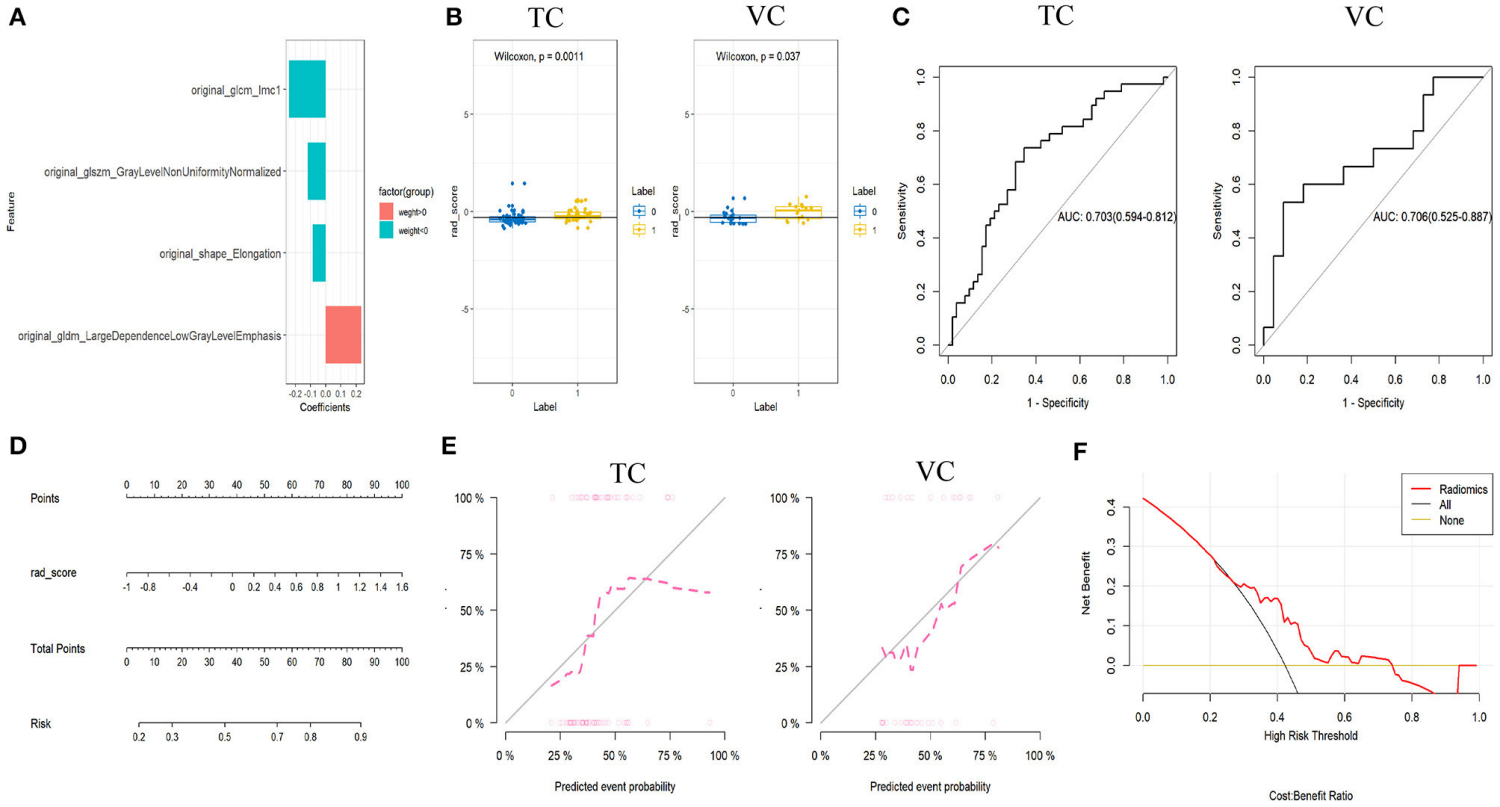
Results of C-ITN patients on the unaffected side and HCs with NVC, analyzed by machine learning. **(A)** Selected features and their importance ratio. **(B)** Wilcoxon test of rad_score in the TC and VC. **(C)** ROC curves of the rad_score model for the TC and VC. **(D)** Nomogram. **(E)** Calibration curves for the TC and VC. **(F)** Decision curve. NVcs, Cisternal segment of CN V; C-ITN, classical trigeminal neuralgia or idiopathic trigeminal neuralgia with neurovascular compression; HCs, healthy controls; TC, training cohort; VC, validation cohort; ROC, receiver operating characteristic curve; NVC, neurovascular compression.

For each ROI of C-ITN and HCs, 117 features were selected. After dimensionality reduction, four textural features with greater weight were identified, namely, size-zone non-uniformity normalized (SZNN), elongation, large dependence low gray-level emphasis (LDLGLE), and informational measure of correlation1 (IMC1; Figure 5).

#### Model construction and evaluation of the rad_score

In both the training and validation cohorts, the Wilcoxon test for the rad_score revealed a statistically significant difference (*P* < 0.05). Logistic regression was employed during the construction of the classifier to select imaging features with greater weight from the cisternal segment of the CN V model. Both cohorts exhibited consistent AUC values, indicating a good model fit (Figure 5).

#### Model construction and evaluation of nomogram

The nomogram model was developed using logistic regression for the rad_score (Figure 5). After constructing the nomogram model, a simplified visual representation of the complex regression equation was created. Calibration curves in the training and validation cohorts demonstrated high diagnostic accuracy of the model (*P* = 0.42 vs. *P* = 0.61). Finally, a decision curve analysis was conducted to assess the clinical utility of the model (Figure 5).

## Discussion

In our study, the majority of C-ITN patients were middle-aged women with right-sided morbidity, and the SCA was identified as the primary offending vessel, consistent with the findings of the previous literature (Zhong et al., 2018; Lee et al., 2019; Bendtsen et al., 2020; Müller et al., 2020; Zhang et al., 2020, 2021; Hung et al., 2021; Inoue et al., 2021; Tohyama et al., 2021; Ge et al., 2022). While 53 CN Vs exhibited NVC on the unaffected side, NVC was present in 74 CN Vs of 78 HCs, suggesting that NVC might not be the sole essential factor for C-ITN but could be a contributing risk factor. The offending vessel, mainly the SCA, mirrored the affected side of C-ITN. However, differences were noted, such as the presence of the VA, BA, and TVHI on the affected side, which were absent on the unaffected side of C-ITN and HCs. NVC on the unaffected side and in HCs predominantly occurred on the left, while the affected side of C-ITN was mainly on the right, indicating that the type of the offending vessel and the side affected with NVC were considered risk factors for C-ITN. In both the affected and unaffected sides of C-ITN and HCs with NVC, the POfvC was typically in the middle and distal two-thirds of the trigeminal cisternal segment, with the DOfvC mainly classified as class 2. However, not all cases with such characteristics exhibited symptoms, suggesting that microstructural changes alongside macrostructural alterations might contribute to symptomatology.

The results indicate that, compared to the clinical model and rad_score model, the nomogram model demonstrated the highest AUC value and better predictive capability for C-ITN occurrence. Furthermore, the side affected by NVC, the type of the offending vessel, and the microstructure of the cisternal segment of CN V (e.g., LAL, CS, range, LAHGLE, contrast, energy, and MDS) were identified as risk factors for unilateral C-ITN. These altered microstructures may stem from varying degrees of degeneration and demyelination.

Machine learning analysis of C-ITN patients on the unaffected side and HCs, both with NVC, revealed that microstructural changes in the cisternal segment of CN V, including SZNN, elongation, LDLGLE, and IMC1, carried greater weight and were identified as risk factors for C-ITN on the side unaffected by NVC. These alterations in texture features may indicate that C-ITN not only affects the CN V on the affected side but also induces changes on the unaffected side.

Although NVC was considered the primary etiology of CTN, its presence alone did not necessarily imply CTN. In our study, NVC was observed on the unaffected sides in 63.10% of C-ITN patients and in 62.82% of HCs. Additionally, 32.05% of HCs had NVC on both sides but remained asymptomatic, indicating that NVC might not be the primary cause of CTN or could only contribute partially to its etiology. Previous studies reported NVC rates of about 25–49% in healthy individuals and 14–39% in autopsy patients, consistent with our findings (Hamlyn, 1997; Kakizawa et al., 2008; Ramesh and Premkumar, 2009; Müller et al., 2020).

In this study, both the C-ITN on the unaffected side and the HCs, both with NVC, did not cause pain symptoms and only occurred in the C-ITN on the affected side, so what are the differences in the structure of the cisternal segment of CN V between them? By comparing the CN V between the affected side and the unaffected side, it was found that the morphology (including cross-sectional area and volume) of the CN V changed more obviously than that of the unaffected side (Lambru et al., 2020; Alper et al., 2021). It was speculated that the cross-sectional area was a risk factor for TN, but there was no study on HCs and TN of those with NVC. Some studies analyzed the microstructure of the CN V based on DTI and found that the FA of the affected side CN V was decreased compared with that of the unaffected side and HCs, while the mean diffusion (MD) and radial diffusivity (RD) were increased in TN patients (Moon et al., 2018; Lee et al., 2019). However, the number of patients included in the study was small and an analysis of the unaffected side CN V based on NVC was not performed. Wang et al. found that the volume of the CN V was reduced in patients with CTN compared with HCs (Wang et al., 2019). Despite no significant difference being observed in the volume of CN V between the affected and unaffected sides in C-ITN patients, the unaffected sides in C-ITN and HCs in this study, it was possible that NVC was present in all the subjects. Thus, these findings provided a more reliable basis for studying the etiology of C-ITN and suggested microstructural differences in the CN V with no significant difference in the macrostructure.

Based on the data presented above, previous studies primarily compared the affected side of TN patients with the unaffected side or with HCs, but most of the studies did not compare the CN V of TN patients with that of HCs who had NVC. In this study, we compared the CN V of C-ITN patients on the affected side with that of HCs and the CN V of C-ITN patients on the unaffected side with that of HCs, both groups having NVC, to lay the groundwork for further investigation into the risk factors or causes of C-ITN. To our knowledge, this was the first study to analyze and investigate the cisternal segment of CN V using the ML method to explore the risk factors of C-ITN, with all subjects having NVC.

In recent years, ML has been increasingly utilized to assist clinicians in decision-making (van Timmeren et al., 2020). ML has been applied in TN research to explore the morphological characteristics of CN V, its pathogenesis, prognostic factors, central changes, and more. Lin et al. (2021) analyzed the flatness feature of Meckel's cave (MC) using the radiomics method and observed an asymmetry in the morphology of bilateral MC in the PTN and HC groups. Mulford et al. (2022) discovered that the texture features and radiomics intensity of the CN V are correlated with the presence of pain from TN. Danyluk et al. (2021) identified several pain-relevant brain regions with an abnormal texture that could differentiate TN patients from HCs. The abovementioned studies used ML in TN research, yet they did not categorize based on the presence of NVC.

In this study, we compared C-ITN patients and HCs based on NVC using ML and found that, compared with HCs, in addition to the affected side CN V, the microstructure of the unaffected side also changed. This study could supplement previous research, which compared the affected and unaffected side CN V of C-ITN patients.

However, this study has some limitations. First, it was a single-center study, which may introduce certain biases, so a multi-center study is needed in future research. Second, the sample size was small, highlighting the necessity to increase the sample size and subgroup based on postoperative effects. Third, the study only analyzed the structure of the cisternal segment of CN V, while future studies should comprehensively examine the trigeminal nerve and its center to identify primary TN risk factors. Finally, we did not compare the ML models with any existing methods or baselines.

## Conclusion

In conclusion, this study revealed that (1) the side of NVC, the type of offending vessel, and the microstructure of the cisternal segment of CN V were risk factors for the pathogenesis of C-ITN; (2) compared with HCs with NVC, there was no significant difference in the macrostructure in the unaffected side of C-ITN with NVC, but there were certain differences in the microstructure, indicating that the unaffected side of C-ITN may be affected to some extent. These data provide a basis for further elucidation of the etiology of C-ITN.

## Data availability statement

The original contributions presented in the study are included in the article/supplementary material, further inquiries can be directed to the corresponding authors.

## Ethics statement

This study was approved by the Ethics Committee of Hangzhou First People's Hospital (IRB# No. 202107002). The studies were conducted in accordance with the local legislation and institutional requirements. Written informed consent for participation was not required from the participants or the participants' legal guardians/next of kin in accordance with the national legislation and institutional requirements. Written informed consent was obtained from the individual(s) for the publication of any potentially identifiable images or data included in this article.

## Author contributions

JY: Writing – original draft, Investigation, Funding acquisition, Data curation. LW: Writing – review & editing, Validation, Software, Methodology, Formal analysis. LP: Writing – review & editing, Visualization, Project administration, Formal analysis, Conceptualization. HY: Writing – review & editing, Validation, Resources, Data curation. XZ: Writing – review & editing, Supervision, Resources, Investigation, Data curation. QF: Writing – review & editing, Validation, Supervision, Methodology, Conceptualization. ZD: Writing – review & editing, Supervision, Conceptualization. XG: Writing – review & editing, Funding acquisition, Data curation. LS: Writing – review & editing, Visualization, Conceptualization.
